# Outcomes of ablative therapy and radical treatment for prostate cancer: a systematic review and meta-analysis

**DOI:** 10.1590/S1677-5538.IBJU.2023.0628

**Published:** 2024-03-10

**Authors:** Guilherme Miranda Andrade, Felipe Giorgi Manente, Pedro José Damato Dias Barroso, Saulo Borborema Teles, Alexandre Dib Partezani, Willy Baccaglini, Rafael Sanchez-Salas, Ruben Olivares, Bruno Nahar, Gustavo Caserta Lemos, Bianca Bianco, Arie Carneiro

**Affiliations:** 1 Hospital Israelita Albert Einstein Departamento de Urologia São Paulo SP Brasil Departamento de Urologia, Hospital Israelita Albert Einstein, São Paulo, SP, Brasil;; 2 Faculdade de Medicina do ABC Centro Universitário Santo André SP Brasil Faculdade de Medicina do ABC - Centro Universitário (FMABC), Santo André, SP, Brasil;; 3 Faculdade Israelita de Ciências da Saúde Albert Einstein São Paulo SP Brasil Faculdade Israelita de Ciências da Saúde Albert Einstein, São Paulo, SP, Brasil;; 4 Institute McGill University Department of Urology Montreal Canada Department of Urology, Institute McGill University, Montreal, Canada;; 5 Case Western Reserve University Cleveland Clinic Lerner College of Medicine Cleveland OH USA Cleveland Clinic Lerner College of Medicine of Case Western Reserve University, Cleveland, OH, USA;; 6 University of Miami Miller School of Medicine Department of Urology Miami USA Department of Urology, University of Miami Miller School of Medicine, Miami, USA

**Keywords:** Prostatic Neoplasms, Minimally Invasive Surgical Procedures, Prostatectomy, Radiotherapy

## Abstract

**Purpose:**

To compare biochemical recurrence, sexual potency and urinary continence outcomes of ablative therapy and radical treatment (radical prostatectomy or radiotherapy with androgen deprivation therapy).

**Material and methods:**

A systematic review and meta-analysis followed the PRISMA guidelines were performed. We searched MEDLINE/PubMed. Biochemical recurrence at three and five years; incontinence rate (patients who used one pad or more) and erectile dysfunction rate at 12 and 36 months (patients who did not have sufficient erection to achieve sexual intercourse) were evaluated. The Mantel-Haenszel method was applied to estimate the pooled risk difference (RD) in the individual studies for categorical variables. All results were presented as 95% confidence intervals (95%CI). Random effects models were used regardless of the level of heterogeneity (I²). (PROSPERO CRD42022296998).

**Results:**

Eight studies comprising 2,677 men with prostate cancer were included. There was no difference in biochemical recurrence between ablative and radical treatments. We observed the same biochemical recurrence between ablative therapy and radical treatment within five years (19.3% vs. 16.8%, respectively; RD 0.07; 95%CI=-0.05, 0.19; *I*^2^=68.2%; P=0.08) and continence rate at 12 months (9.2% vs. 31.8%, respectively; RD −0.13; 95%CI, −0.27, 0.01; *I*^2^=89%; P=0.32). When focal treatment was analyzed alone, two studies with 582 patients found higher erectile function at 12 months in the ablative therapy group than in the radical treatment (88.9% vs. 30.8%, respectively; RD −0.45; 95%CI −0.84, −0.05; *I*^2^=93%; P=0.03).

**Conclusion:**

Biochemical recurrence and urinary continence outcomes of ablative therapy and radical treatment were similar. Ablative therapy appears to have a high rate of sexual potency.

## INTRODUCTION

Prostate cancer (PCa) is the second most common cancer in men, responsible for 15% of all malignant tumors (1). The 2023 American Urological Guidelines recommend screening for PCa aiming to reduce cancer-related mortality (2). Concomitant with early diagnosis, there has been an increase in the treatment. Standard treatment options for primary PCa include active surveillance, radical prostatectomy (RP), radiotherapy (RT), and brachytherapy. These interventional treatments have limitations, such as intraoperative bleeding, radiation injury, and injury to the surrounding tissues (3, 4).

In this context, novel focal treatments such as ablative therapy (AT) have emerged as alternatives to whole-gland radical treatments, aiming to reduce treatment-related toxicity by sparring prostatic tissue as much as possible (5). The most popular AT options include high-intensity focused ultrasound (HIFU), cryotherapy, irreversible electroporation, photodynamic therapy, and focused laser ablation (6).

Several cohorts and trials have compared ATs. Recent reviews have focused on either one specific AT or nonsurgical salvage treatment instead of the primary treatment for PCa (6, 7). The limited scope of previous reviews and recent publications assessing multiple ATs options and comparing oncology outcomes between ATs and standard treatments require a new and comprehensive meta-analysis (8).

The hypothesis is that patients who receive ablative treatment for PCa may have functional benefits regarding urinary continence and sexual potency compared to patients undergoing standard treatments, with the same oncological outcomes in both groups. This study aimed to compare biochemical recurrence (BCR), urinary incontinence, and erectile dysfunction rates after AT and radical treatment (RAD) through a systematic review and meta-analysis.

## MATERIAL AND METHODS

### Systematic review and meta-analysis

This study was based on the PRISMA statement (9) and registered in the International Prospective Register of Systematic Reviews (PROSPERO) (number CRD42022296998).

Studies that included at least one ablative therapy (e.g., HIFU alone or HIFU plus cryotherapy) were included. RP or RT with androgen deprivation therapy were included in the control group. Studies should assess at least one of the following outcomes: biochemical recurrence rate (BCR), urinary incontinence rate, or erectile dysfunction rate.

### Search strategy and selection criteria

We systematically searched the MEDLINE/PubMed database for articles published in English until July 2023, including participants with prostate cancer undergoing ablative treatment (cryotherapy, HIFU, Tookad, laser ablation, photodynamic therapy, and irreversible electroporation) or radical treatment (radical prostatectomy and radiotherapy).

Comparative studies were included between ablative therapy (experimental group) and radical treatment (control group) in patients with localized PCa. All types of focal therapy were considered for the first analysis and all techniques (such as whole gland, hemigland, or actual focal ablation).

A systematic search was conducted using the "Clinical Trial," "Meta-Analysis," "Randomized Controlled Trial," "Systematic Review" and the following keywords ("prostate cancer") AND ("cryotherapy" OR "cryosurgery" OR "HIFU" OR "high intensity ultrasound" OR "tookad" OR "laser ablation" OR "photodynamic therapy" OR "irreversible electroporation" OR "focal ablation").

For evaluation of oncologic success, we used the BCR as criteria at three and five years using the Phoenix criteria (consecutive PSA greater than 0.2 ng/mL after radical prostatectomy or focal therapy, and for post-radiotherapy cases, it was defined when the PSA value was above the PSA Nadir +2). Regarding the functional analysis, the incontinence rate at 12 and 24 months (patients who used one pad or more) and erectile dysfunction rate at 12 and 36 months (patients who did not have sufficient erection to achieve sexual intercourse) were evaluated.

### Data extraction

Two authors (GMA. and FGM.) independently searched for and selected articles. In case of discordance, a third author (SBT) resolved the differences.

The following baseline data were collected for each study: mean age, prostate volume (cm3), PSA level (ng/mL), T stage, Gleason score, and/or International Society of Urological Pathology (ISUP) grade, number of positive and total cores, type of AT (HIFU, cryotherapy, HIFU plus cryotherapy), and extension (whole gland, hemigland, focal therapy) of intervention in the experimental group, and type of intervention in the control group (RP or R).

### Data Synthesis

The Mantel-Haenszel method was applied to estimate the pooled risk difference (RD) in the individual studies for categorical variables. All results were presented as 95% confidence intervals (95% CI). Random effects models were used, regardless of the level of heterogeneity (I²). We used Review Manager version 5.4 (*The Cochrane Collaboration*, London, England, UK). Statistical significance was set at P< 0.05.

### Risk of bias assessment

The quality assessment was performed with AMSTAR 2 for systematic reviews (10), the Cochrane Collaboration tools for risk of bias RoB-2 for randomized controlled (11) and RoB-I for other study types (12), ranging from high, unfavorable or unclear, moderate and low quality.

## RESULTS

### Study selection and characteristics

Eight studies were included in this meta-analysis, enrolling 2,677 patients, being three randomized trials and five were retrospective studies. Regarding the risk group of patients included in each study, we had a predominance of intermediate-risk patients in five of the eight studies. In the other three studies, there was a prevalence of high-risk patients in two studies and a prevalence of low-risk patients in only one study. Therefore, the majority of patients included in this meta-analysis were of intermediate risk, with the considerations presented in the discussions being relevant mainly for this group of patients.

**Figure 1:**
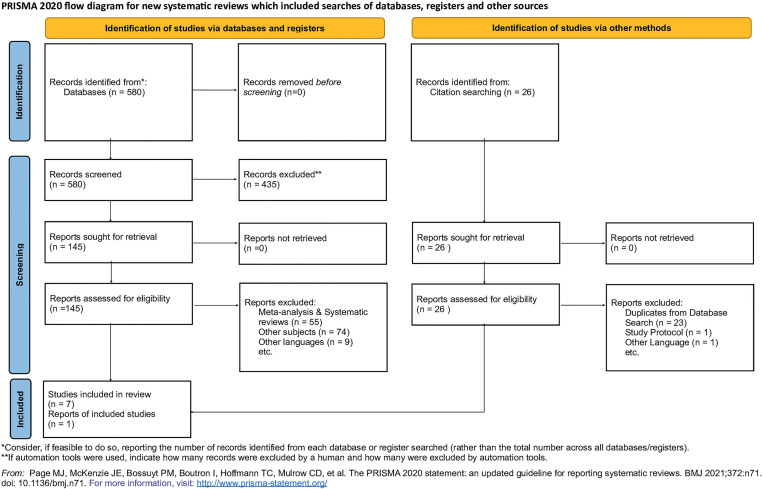
Flow diagram of included studies (PRISMA flow).

Figure-1 presents a PRISMA flowchart of the studies included in the systematic review. A summary of this evidence is presented Table-1 (13–20).

**Table 1:** Summary of evidence of studies included.

Study (Year)	Groups	N	Treatment	Age a	PSA b	Biochemistry recurrence			Sexual Function			Continence			Risk of Bias (Final Judgement)
						36 mo	60 mo	Criteria	12 mo	36 mo	Criteria	12 mo	24 mo	Criteria	
**Randomized Clinical Trials**															
Robinson et al. (2009) (13)	Group 1	117	Cryoblation	69.4	8.1	-	-	-	9/34	10/34	Erections sufficient for sexual intercourse	-	-	-	Moderate quality
	Group 2	114	Radiotherapy	68.6	9.0	-	-	-	24/30	18/30	Erections sufficient for sexual intercourse	-	-	-	
Donelly et al. (2010) (14)	Group 1	117	Cryoblation	69.4	8.1	20/117	29/117	PSA nadir+ 2 ng/dL	-	14/56	Erections sufficient for sexual intercourse	-	-	-	Moderate quality
	Group 2	114	Radiotherapy	68.9	9.0	15/114	29/114	PSA nadir+ 2 ng/dL	-	15/57	Erections sufficient for sexual intercourse	-	-	-	
Chin et al. (2012) (15)	Group 1	31	Cryoblation	70.4	11.1	17/31	22/31	PSA nadir+ 2 ng/dL	-	-	-	-	-	-	Moderate quality
	Group 2	31	Radiotherapy	70.5	8.6	6/31	7/31	PSA nadir+ 2 ng/dL	-	-	-	-	-	-	
**Retrospective Studies**															
Albissini et al. (2017) (16)	Group 1	55	HIFU (hemigland)	73.0	6.9	7/55	-	PSA nadir+ 2 ng/dL	24/30	-	Erections sufficient for sexual intercourse	52/55	52/55	0 pads	Moderate quality
	Group 2	55	Radical Prostatectomy	63	6.5	6/55	-	PSA ≥ 0.2 ng/dL	27/48	-	Erections sufficient for sexual intercourse	48/55	50/55	0 pads	
Chinevovo et al. (2018) (17)	Group 1	42	cryoablation	69.0	6.5	-	-	-	-	-	-	42/42	-	0 pads	Moderate quality
	Group 2	42	Radical Prostatectomy	65.0	6.7	-	-	-	-	-	-	45/42	-	0 pads	
Garcia-Barreras et al. (2018) (18)	Group 1	236	HIFU and cryoablation	68.2	7.12	21/336	-	PSA nadir+ 2 ng/dL	145/160	-	Erections sufficient for sexual intercourse	208/236	-	0 pads	Moderate quality
	Group 2	472	Radical Prostatectomy	65.1	6.99	29/472	-	PSA ≥ 0.2 ng/dL	94/344	-	Erections with or without PDE5i	300/472	-	0 pads	
Rosenhammer et al. (2019) (19)	Group 1	365	HIFU	67.5	7.1	45/365	62/365	PSA nadir+ 2 ng/dL	-	-	-	-	-	-	Low quality
	Group 2	394	Radical Prostatectomy	62.8	9.2	37/394	52/394	PSA ≥ 0.2 ng/dL	-	-	-	-	-	-	
Shah et al. (2021) (20)	Group 1	246	HIFU and cryoablation	63.3	7.9	22/246	34/246	Gleason ≤7 in biopsy	-	-	-	-	-	-	Moderate quality
	Group 2	246	Radical Prostatectomy	63.4	7.9	24/246	44/246	PSA ≥ 0.2 ng/dL	-	-	-	-	-	-	

a Median years old;

b PSA level (ng/mL) before treatment;

mo = months; PDE5i = Phosphodiesterase 5 inhibitor.

### Risk of bias assessment

**Supplementary Figure 1:**
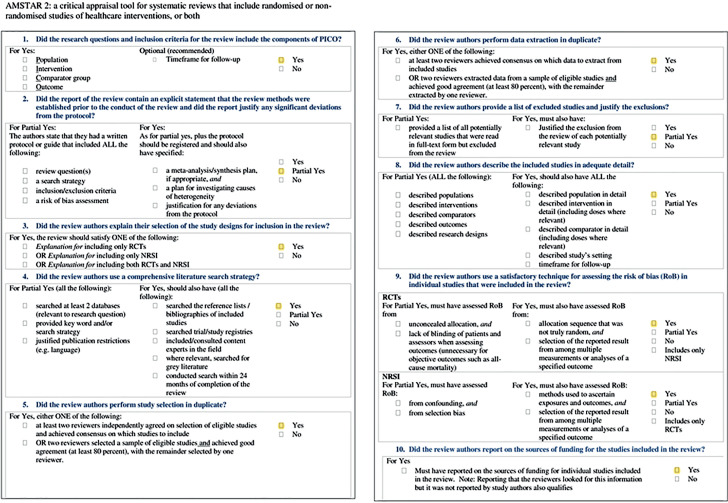
AMSTAR 2.

**Supplementary Figure 2:**
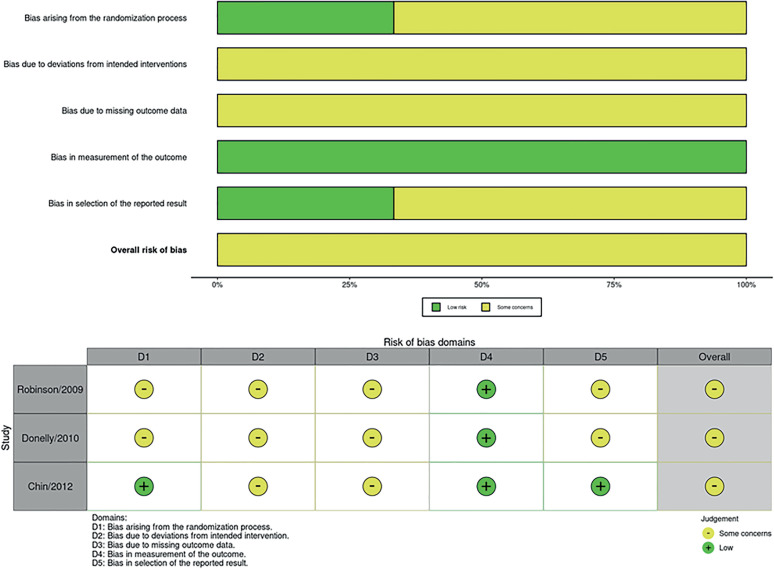
Rob 2.

**Supplementary Figure 3:**
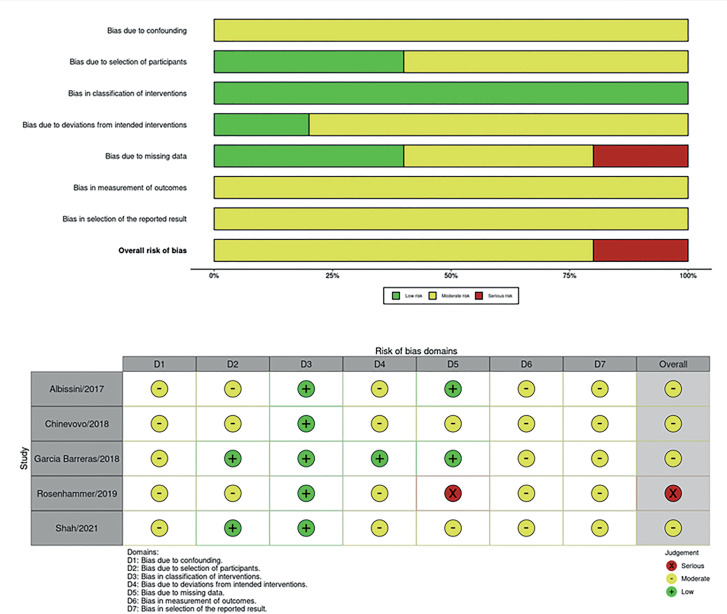
Rob-1.

Using the AMSTAR 2 Guidance Document was possible to identify moderate confidence in the results of the review (Supplementary Figure-1). Three randomized trials were assessed using the Rob-2 tool (Supplementary Figure-2). Three studies had concerns regarding the risk of bias, which was characterized as moderate quality. The main deviations were observed in the randomization process, outcome measurements, and selection of reported results. Retrospective studies were assessed using the Rob-1 tool and rated as moderate (four studies) and low quality (one study) (Supplementary Figure-3).

### Results of syntheses - Meta-analysis

#### Biochemical recurrence

**Figure 2:**
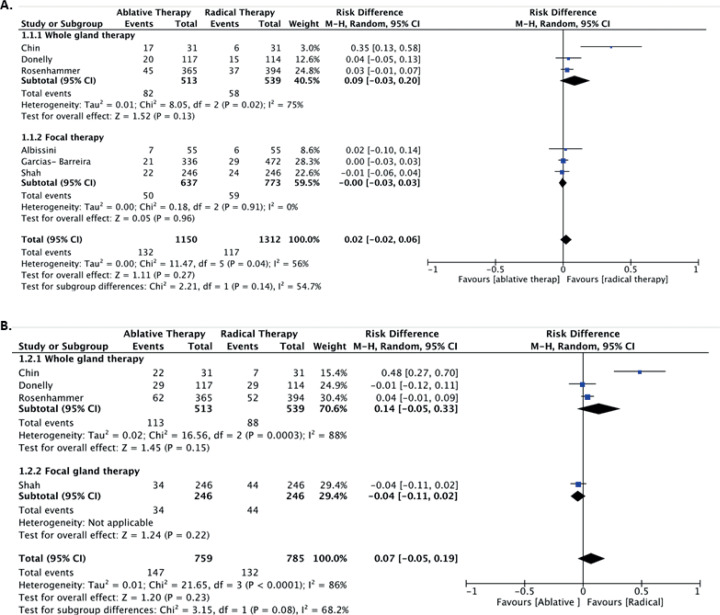
Biochemical recurrence in 3 years (A) and 5 years (B).

Regarding BCR, six studies were included with a total of 2,462 patients (14–16, 18–20). Only Shat et al. (20) adopted a systematic biopsy after local treatment instead of following the patient's PSA levels (Gleason 7 or above was used to determine oncological failure) in the AT arm. The comparison of BCR at three years between the experimental (AT) and control (RAD) groups was 11.4% and 8.9%, respectively (RD 0.02; 95% CI =-0.02, 0.06; I2=56%; P=0.14). Considering only the studies on whole gland therapy (14, 15, 19), the results were also similar (RD 0.09, 95%, CI=-0.03, 0.20, I2=75%; P=0.13). Analysis of only focal treatment (16, 18, 20) revealed similar BCR between AT and RAD (RD 0.00, 95%, CI= −0.03, 0.03, I2=0%, P=0.96) (Figure-2A).

The comparison of BCR at five years, considering four out of six studies comprising 1,544 patients (14, 15, 19, 20), between AT and RAD was 19.3% versus 16.8%, respectively (RD 0.07; 95% CI=-0.05, 0.19; I2=68.2%; P=0.08). Considering only whole gland studies, we also did not find any difference between AT and RAD (RD 0.14; 95% CI= −0.05, 0.33; I2=88%; P=0.15). Shah et al. (20) analyses BCR between focal therapy and RAD and did not find a difference between the groups in five years (Figure-2B).

#### Incontinence rate

**Figure 3:**
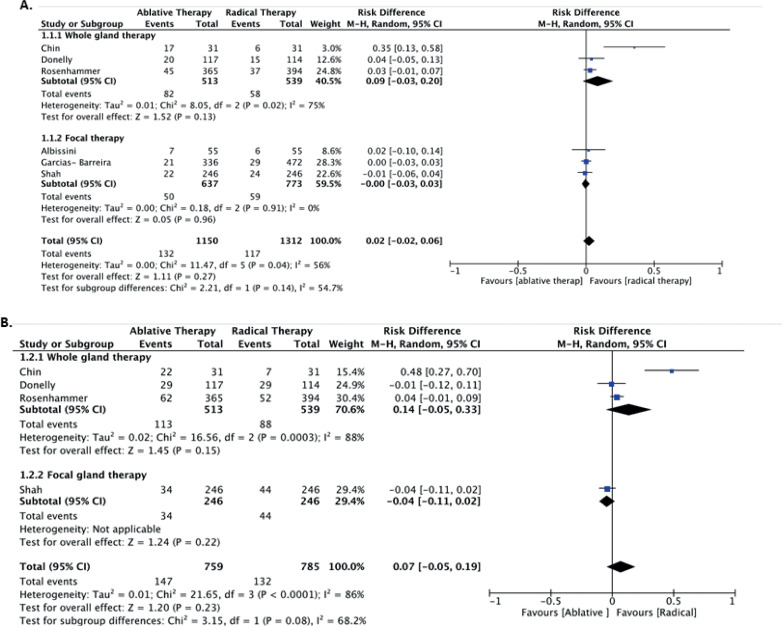
Continence rate in 12 months (A), and sexual potency in 12 and 36 months (B).

Three studies including a total of 908 patients assessed the incontinence rate between both groups (16–18). A trend in favor of AT at 12 months was observed, although it was not statistically significant (9.2% vs. 31.8%, respectively; RD −0.13; 95% CI, −0.27, 0.01; I2=89%; P=0.32). The sensitivity analysis considering the whole gland and focal treatment also did not show any statistical difference between AT and RAD (Figure-3A).

#### Sexual function

Four studies including 759 patients assessed erectile function between AT and RAD (13, 14, 16, 18). Considering both whole gland and focal treatment versus RAD, there was no difference in potency (RD −0.08; 95% CI, −0.62, 0.45; I2=98%; P=0.03). When only focal treatment was considered, two studies (16, 18) with 582 patients found lower erectile dysfunction at 12 months in the AT group than in the RAD (11.1% vs. 69.2%, respectively; RD −0.45; 95% CI=-0.84, −0.05; I2=93%; P=0.03) (Figure-3B). The comparison of sexual function between AT and RAD at 36 months included only two studies (13, 14), with no difference between them (RD 0.15, 95%, CI= − 0.14, 0.43, I2=76%, P=0.31).

## DISCUSSION

To the best of our knowledge this is the first meta-analysis to compare urinary continence and sexual potency rates after focal therapy with radiotherapy or radical prostatectomy, despite there were some systematic reviews in the literature (21–24). We observed better results of focal therapy regarding sexual potency, and although focal therapy presented better average rates for urinary incontinence it carried no statistical significance.

Despite the significant growth in recent years, focal therapy remains a controversial issue. Among the most used focal therapies, the following stand out: HIFU, electroporation, cryotherapy, photodynamic therapy and focal laser ablation (25–27). The main concerns about the use of focal therapy in prostate cancer treatment are the risk of cancer recurrence and the functional outcomes (sexual potency and urinary continence).

The present study showed that AT resulted in satisfactory oncologic control, similar to RAD outcomes, with BCR rates of 11.4% and 19.3% at three and five years, respectively. Regarding functional outcomes, the urinary incontinence rates at 12 and 24 months were about two times lower than those observed in the RADs group, however, with no statistical significance. The potency preservation rate was higher in focal ablative therapy.

Studies on ablative therapies have a great variety of methodologies and analyzed different outcomes, leading to a high rate of heterogeneity in the results (21, 22). Furthermore, it is of paramount importance to distinguish the type of energy used in ablative therapy, technology, and ablation performed, which are the determining factors for the result. Thus, making joint assessment of data difficult, only a small number of articles were included in this meta-analysis.

The oncological success is quite heterogeneous, with the most used outcome being BCR (the majority used the Phoenix criterion), presence of clinically significant PCa in the control biopsy, and the rate of need for rescue treatment. Ideally, all patients undergoing prostate ablative treatment should be re-evaluated 6-12 months after biopsy to control the effectiveness of treatment. However, this is challenging in the clinical practice, in many cases as PSA levels decrease significantly the patients do not want to undergo an invasive procedure. As a result, in many series the sample of patients who underwent biopsy may also be contaminated, as they may include a large proportion of patients whose PSA levels did not decrease or showed some alteration in the control MRI. Therefore, the assessment of oncologic success remains a challenge, and BCR is most commonly used in publications.

This review showed that the BCR rate varied from 8-54% in AT and from 6-19% in RAD. At three and five years the rates of BCR were similar. However, comparative studies considering a control biopsy and a longer follow-up time should be conducted to better understand the real oncological impact of the treatments.

Detailing each modality of focal therapy, Albissini et al. (16) showed that HIFU is comparable to radical prostatectomy regarding the success of oncological outcomes, that is, the need for rescue therapy (16, 23). Similar oncological outcomes were also observed by Donnelly et al., Chinenov et al., and Shah et al., with the advantage of being less invasive and presenting less impact in patients’ quality of life (14, 17, 20). Nevertheless, Chin et al. found worse oncological outcomes with focal therapy (cryoablation) than with radiotherapy (15). BCR after standard and focal therapies was not significantly different in this meta-analysis.

Regarding functional outcomes, the present study corroborates the current literature on better AT results than RAD. The difference in the potency rate over one year was about 58% (11.1% vs. 69.2%), indicating that AT is clinically and statistically superior to RAD. It is important to consider the high heterogeneity and the small number of studies included as a bias factor in this analysis.

In the literature, the urinary incontinence rate in AT ranges from 5% to 11% and in RAD from 12% to 36% (16–18, 28), corroborating our findings. Although not statistically different, in this present study it was observed lower rates of urinary incontinence in the AT group (9.2% vs. 31.8%).

In agreement with Tay et al. (27), we observed that ablative therapy is a safe treatment with low levels of impairment of sexual function and urinary continence. Albissini et al. (16) observed that HIFU presented better functional outcomes than standard therapy. Garcia-Barreras et al. (18) also showed that focal treatment methods have an advantage in terms of functional results compared to radical prostatectomy. On the other hand, Robinson et al. reported a minor advantage of radiotherapy over cryoablation preserving sexual potency (13).

This study has some limitations. The evaluation of urinary incontinence according to the use of pads makes a categorical variable (yes or no) and disregards different levels of urinary incontinence or overestimates dry patients who use PAD for safety. In addition, the evaluation time of most studies was only 12 months; thus, patients who recovered continence after this period were considered incontinent in the 12-month evaluation. Moreover, the use of BCR for oncological success remains controversial. The small number of studies resulted in high heterogeneity in the analysis; thus, the findings should be interpreted with caution. Besides, randomized controlled trials are necessary to confirm the findings.

## CONCLUSION

Oncological outcomes between RAD and AT modalities of treatment presented similar results considering the BCR at three and five years. Focal therapy was associated with higher rates of erectile function at 36 months. Considering the current data available in the literature, the findings suggested that focal therapy may be offered to well-selected patients to avoid or delay RAD. Prospective and randomized studies with standardized outcomes should be conducted to consolidate these concepts and to validate AT as a standard modality for prostate cancer treatment.
